# The Association Between HIV-Related Stigma and the Uptake of HIV Testing and ART Among Older Adults in Rural South Africa: Findings from the HAALSI Cohort Study

**DOI:** 10.1007/s10461-023-04222-w

**Published:** 2024-01-29

**Authors:** Nomsa B. Mahlalela, Jennifer Manne-Goehler, Daniel Ohene-Kwofie, Leslie B. Adams, Livia Montana, Kathleen Kahn, Julia K. Rohr, Till Bärnighausen, Francesc X. Gómez-Olivé

**Affiliations:** 1 MRC/Wits Rural Public Health and Health Transitions Research Unit (Agincourt), Faculty of Health Sciences, School of Public Health, University of the Witwatersrand, Johannesburg, South Africa; 2 Health Economics and Epidemiology Research Office, Faculty of Health Sciences, University of the Witwatersrand, Johannesburg, South Africa; 3 Medical Practice Evaluation Center, Massachusetts General Hospital, Harvard Medical School, Boston, MA USA; 4 Division of Infectious Diseases, Brigham and Women’s Hospital, Harvard Medical School, Boston, MA USA; 5 Center for Population and Development Studies, Harvard University, Cambridge, MA USA; 6 Department of Mental Health, Johns Hopkins Bloomberg School of Public Health, Baltimore, MD, USA; 7 The DHS Program, ICF, Rockville MD, USA; 8 Heidelberg Institute of Global Health, University of Heidelberg, Heidelberg, Germany; 9 Africa Health Research Institute (AHRI), Mtubatuba, South Africa

**Keywords:** HIV-related stigma, Antiretroviral therapy, HIV testing, Older adults, South Africa, HAALSI

## Abstract

HIV testing and antiretroviral therapy (ART) remain critical for curbing the spread of HIV/AIDS, but stigma can impede access to these services. Using data from the Health and Aging in Africa: A Longitudinal Study of an INDEPTH Community in South Africa (HAALSI), we used a multivariable logistic regression to examine the correlation between HIV-related stigma, HIV testing and ART uptake in older adults. We used four questions to measure stigma, with three assessing social stigma (reflecting social distancing preferences) and one assessing anticipated stigma (disclosure concern). We combined the three social stigma questions to generate a social stigma score ranging from 0 to 3, with higher scores indicating higher stigma. Anticipated stigma was prevalent 85% (95% CI 0.84–0.86), and social stigma was also frequent 25% (95% CI 0.24–0.27). Higher social stigma scores correlated with decreased HIV testing for all participants with social stigma. Compared to those with a score of 0, odds of testing decreased with higher stigma scores (OR = 0.66, 95% CI 0.53–0.81, p = 0.000) for a score of 1 and (OR = 0.56, 95% CI 0.38–0.83, p = 0.004) for a score of 3. ART uptake also decreased with higher social stigma scores among people living with HIV (PLWH), although it was significant for those with a score of 2 (OR = 0.41, 95% CI 0.19–0.87, p = 0.020). These findings emphasize that HIV-related stigma hampers testing and ART uptake among older adults in rural South Africa. Addressing stigma is crucial for improving testing rates, early diagnosis, and treatment initiation among the older population and achieving UNAIDS 95–95–95 targets.

**Supplementary Information:**

The online version contains supplementary material available at 10.1007/s10461-023-04222-w.

## Introduction

The HIV/AIDS epidemic remains a significant public health challenge globally, with approximately 39 million people living with HIV (PLWH), and 1.3 million new infections in 2022 [1]. South Africa continues to have a high HIV prevalence of 13.7% among all age groups, accounting for about 8.2 million PLWH [2]. Notably, the number of PLWH aged 50 and older is increasing globally, indicating an aging HIV-positive population [3]. Evidence from rural South Africa shows high HIV prevalence among older adults [4–6], attributed to increased life expectancy among PLWH on antiretroviral therapy (ART) and new infections occurring at older ages [3, 6–8]. Older adults are a growing at-risk group for HIV transmission, and their inclusion in HIV prevention and care treatment programs is crucial for reaching the UNAIDS 95–95–95 targets to combat the HIV epidemic.

Despite the decline in sexual activity with age, evidence from various countries including South Africa indicates that a significant number of older adults aged 50 and above are sexually active and engage in similar HIV risk behaviors as young people [9–15]. Studies have shown lack of condom use among many older adults with multiple sexual partners [12, 13, 16]. It is crucial for older people to understand HIV transmission risks and be actively included in prevention strategies and HIV services [11]. Early diagnosis and treatment are essential components of the global HIV response to prevent further HIV transmissions [17]. Globally, there has been a significant improvement across the HIV testing and treatment cascade. Among all PLWH, 86% knew their status, 76% were accessing treatment and 71% were virally suppressed in 2022 [1]. While South Africa has made progress in achieving the UNAIDS 90–90–90 targets [18], there are still challenges in linking people to care and retaining them in treatment. Testing rates also remain low among certain high-risk populations, including older adults, despite efforts to expand access to HIV testing and treatment. Community and home-based outreach HIV testing programs aimed at reducing late diagnoses have revealed inadequate testing among older populations due to misconceptions about their sexual activity, lower perception of HIV risk and presence of HIV-related stigma [19, 20].

HIV-related stigma encompasses negative beliefs, feelings and attitudes towards PLWH, groups associated with PLWH such as their families, and key populations at higher risk of HIV infection, such as sex workers, men who have sex with men, transgender people, and people who inject drugs [21]. HIV-related stigma can manifest in various forms, including social stigma (negative attitudes towards PLWH including desires for social distance), enacted stigma (specific acts of discrimination towards PLWH), internalized stigma (negative beliefs within PLWH about themselves) [22], and anticipated stigma (fear of negative outcomes if HIV status is disclosed) [22–24]. These forms of stigma can result in social exclusion, discrimination, rejection, and fear of disclosing HIV status.

HIV remains a highly stigmatized condition because it is often linked to socially condemned behaviours that are considered high risk and associated with its transmission, such as multiple and concurrent sexual partners, homosexual intercourse, drug use and sex work [25–27]. As a result of this association, PLWH often face discrimination and prejudice which often contribute to the ongoing stigma surrounding HIV. Fear and misinformation about HIV transmission also play a significant role in reinforcing unfounded fears of casual contact with PLWH. This perpetuate harmful myths about HIV, leading to the continuation of stigma associated with the virus. HIV-related stigma often intersects with other forms of stigmatization such as homophobia, gender discrimination, discrimination against marginalized populations, and racism further creating complex challenges for PLWH. Intersecting stigmas can result in social exclusion, limited access to healthcare and support, increased psychological distress, and reduced opportunities for employment and personal relationships.

HIV-related stigma remains a significant barrier to controlling the HIV epidemic. Stigmatizing attitudes discourage individuals from getting tested for HIV, seeking medical care, and adhering to treatment [7, 28–32]. Fear of stigma can prevent people from disclosing their HIV status to family, friends, or sexual partners, leading to increased feelings of isolation and secrecy. Stigma also affects mental health and overall well-being for PLWH, and present challenges for those in need of health care services including older adults [32]. Overcoming stigma requires comprehensive strategies that promote education, awareness, and empathy, fostering a supportive environment and understanding society.

There is a relatively small number of studies on HIV-related stigma among older adults, despite the increasing number of older adults living with HIV. The majority of research excludes older subjects or ignores age as a variable [7]. Older adults experienced the HIV epidemic differently, with historical perspectives that may influence their experiences of stigma. HIV-related stigma has been linked to adverse psychological and social outcomes, which may be particularly important for older adults due to their increased vulnerability. Understanding HIV-related stigma in older adults remains crucial and can inform interventions to support their mental health and overall well-being. This study aims to examine the association between HIV-related stigma and HIV testing and ART uptake among older adults living with HIV in rural South Africa, using data from the HAALSI study.

## Methods

### Study Site

The study was conducted in the Agincourt Health and Demographic Surveillance System (HDSS) site located in Mpumalanga province, South Africa, which is run by the MRC/Wits Rural Public Health and Health Transitions Research Unit. The HDSS conducts an annual census and collects vital events data for all household members in the area, including births, deaths, and migration [33]. Overall, Agincourt is a rural area with underdeveloped education system, limited access to employment opportunities, insufficient healthcare and sanitation, and experiences high rates of labor migration [34, 35].

### Study Population

The HAALSI study is a longitudinal population-based cohort study focused on studying health, aging, and well-being of older people [36]. The baseline assessment data were collected between November 2014 and November 2015. To be included in the study, participants needed to be at least 40 years old as of 1 July 2014 and have lived in the study area for the 12 months preceding the 2013 HDSS census round. A random sample of eligible men and women was obtained from the HDSS database, resulting in a total of 5059 participants (2345 men and 2714 women). Data collection involved in-person interviews conducted by locally trained fieldworkers in the local language (Xi-Tsonga) using a computer-assisted personal interviewing (CAPI) system. Additional details are described elsewhere [36]. Follow-up interviews were conducted from 2018 to 2019 (wave 2). During this period, 602/5059 participants died, leaving 4457 participants eligible for follow-up. Out of these, 4176 participants completed the follow-up interviews, resulting in a response rate of 94%. For the analysis of HIV-related stigma, 3849 participants (92%) responded to all three social stigma questions and were eligible for inclusion. Participants with missing responses (n = 10) or those who refused to answer the stigma questions (n = 317) were excluded from the analysis because we could not generate a social stigma score for them.

### Measures

#### HIV-Related Stigma

HIV-related stigma was assessed using a standard indicator commonly used in UNAIDS general population surveys, Demographic and Health Surveys (DHS) AIDS module, and Family Health International Behavioral Surveillance Surveys (FHI BSS) [37]. The indicator consists of three questions assessing social stigma (desires for social distance from PLWH): (1) “If a member of your family became sick with the AIDS virus, would you be willing to care for him or her in your household?”, (2) “If you knew that a shopkeeper or food seller had the AIDS virus, would you buy fresh vegetables from them?”, (3) “If a female teacher has the AIDS virus but is not sick, should she be allowed to continue teaching in school?”. Negative responses (No) to these questions indicate a preference for social distancing from PLWH [38]. Similar to [39], we defined a respondent as having preferences for social distancing if he/she had a negative response to at least one of the three questions. Each negative response was given a score of one point, and the scores from the three questions were summed to create an overall social stigma score ranging from 0 to 3, with higher scores indicating higher levels of social stigma. The indicator also includes one question assessing anticipated stigma (disclosure concern), “If a member of your family became infected with the AIDS virus, would you want it to remain a secret?.” Positive responses (Yes) to this question reflect the fear of disclosing a hypothetical HIV infection within a family [40], or fear of negative consequences such as rejection or condemnation, if a family member’s HIV positive status were revealed to others [41].

#### Ever Tested for HIV

During wave 2 data collection, participants were asked whether they had ever tested for HIV, with response options of “Yes” and “No”. Participants with missing responses (n = 12) were excluded from the analysis. To ensure accurate estimation of HIV testing rates and to avoid potential bias due to over reporting, participants with a confirmed HIV diagnosis from the dried blood spots (DBS) testing (n = 715) were also excluded from this specific analysis.

#### HIV and ART Uptake Status

During the in-person home interviews, DBS samples were collected from the study participants. After the data collection period, these DBS samples underwent biological testing for various parameters related to HIV. The testing included checking for the presence of HIV antibodies, measuring viral load, and assessing exposure to emtricitabine (FTC) or lamivudine (3TC), which are components of the first- and second-line ART regimens used in the South African HIV program [36, 42].

### Data Analysis

The analysis was conducted using Stata 17 [43]. Descriptive statistics were used to describe the characteristics of the study population. To assess respondent’s attitudes towards PLWH, the proportion of men and women answering “yes” to at least one of the three social stigma questions was calculated. Pearson’s chi-square tests were used to compare proportions. Means in social stigma were calculated using the social stigma score for key socio-demographic groups and the three major “HIV cascade” groups: HIV-negative, HIV+ on ART, and HIV+ no ART, and compared differences between two means using t-tests and three means using one-way ANOVA where applicable. Descriptive analysis was conducted to assess differences in anticipated stigma by key several socio-demographics. Logistic regression analysis was used to examine the association between the outcome of interest (ever tested for HIV) and our independent variables (social stigma and anticipated stigma), while controlling for several confounding variables such as sex, age, education, marital status, employment, household size, and wealth asset index. The same model was applied for the outcome of ART uptake for PLWH. Models were further stratified by age and sex to explore associations in these subgroups. A significance threshold of p < 0.05 was used to determine statistical significance in all analyses.

## Results

The study analysed sample characteristics by sex and included 3,849 participants, with 56% being women (see Table 1). The overall mean age of the participants was 64.6 years (SD = 12.2). Women had lower levels of education (47.5% vs 38.2%, χ^2^ (2) = 39.778, p = 0.000) and more frequently reported being widowed (49.6% vs 14.7%, χ^2^ (3) = 560.617, p = 0.000), while men were more likely to be currently married or living with a partner (63.7% vs 32.5%, χ^2^ (3) = 560.617, p = 0.000). Unemployment was higher among women compared to men (63.0% vs 54.2%, χ^2^ (2) = 35.179, p = 0.000). Men had a higher percentage of living in single-member households (15.5% vs 7.7%, χ^2^ (3) = 63.645, p = 0.000) and were more likely to live in households ranked lowest in the wealth asset index (28.0% vs 23.6%, χ^2^ (4) = 12.022, p = 0.017). Uptake of HIV testing was similar for both men and women based on self-report, and although not significant, HIV prevalence was slightly higher for women 24.6% compared to men 23.9% based on the DBS HIV test results.

**Table 1:** Socio-demographic characteristics by sex among HAALSI participants (N = 3849)

Variables	Total %	% Men (n = 1693)	% Women (n = 2156)	Chi-square (χ^2^)	p value
Age group				χ^2^ (4) = 8.290	0.082
40–49	12.4	12.3	12.4		
50–59	25.6	23.5	27.3		
60–69	28.6	29.7	27.8		
70–79	20.7	21.7	19.9		
80+	12.7	12.9	12.6		
Mean age	64.6	65.1	64.3		
Education				χ^2^ (2) = 39.778	0.000
No formal	43.4	38.2	47.5		
At least some primary (1–7 years)	35.3	36.8	34.0		
Secondary or more (8+ years)	21.3	25.0	18.5		
Marital status				χ^2^ (3) = 560.617	0.000
Never married	7.2	9.6	5.4		
Separated or divorced	12.3	12.1	12.5		
Widowed	34.2	14.7	49.6		
Currently married or living with partner	46.2	63.7	32.5		
Employment status				χ^2^ (2) = 35.179	0.000
Unemployed	59.1	54.2	63.0		
Employed (full-time or part-time)	17.0	20.3	14.4		
Retired	23.9	25.5	22.6		
Household size				χ^2^ (3) = 63.645	0.000
Living alone	11.1	15.5	7.7		
Living with another person	10.4	10.9	9.9		
Living in 3–6 person	43.7	40.6	46.2		
Living in 7+ person	34.8	33.0	36.2		
Wave 2 wealth asset index				χ^2^ (4) = 12.022	0.017
Index 1 (lower)	25.5	28.0	23.6		
Index 2	26.5	24.5	28.1		
Index 3	6.1	6.1	6.2		
Index 4	13.6	13.4	13.8		
Index 5 (higher)	28.2	28.1	28.4		
Wave 2 ever tested for HIV (self-report)				χ^2^ (1) = 0.163	0.687
Yes	76.9	76.6	77.2		
No	23.1	23.4	22.8		
Wave 2 DBS HIV test result				χ^2^ (2) = 1.193	0.551
HIV+	24.3	23.9	24.6		
HIV−	75.2	75.7	74.8		
Indeterminate	0.5	0.4	0.7		

Missing data: Ever tested for HIV (n = 12), Wave 2 DBS HIV test result (n = 902)

**Fig. 1:**
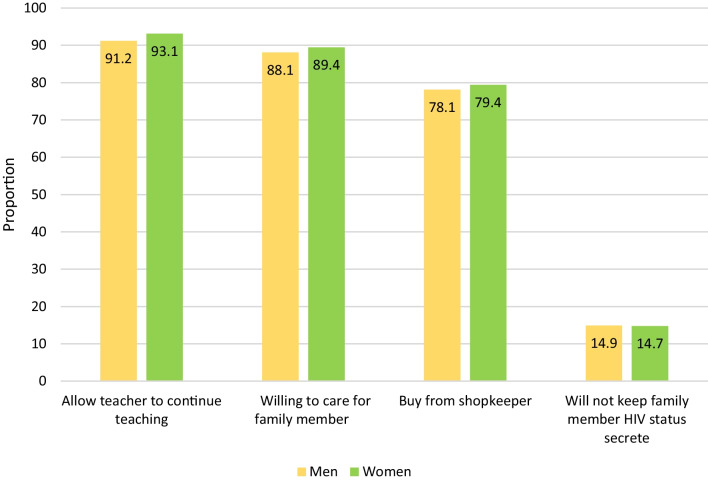
Accepting attitudes toward PLWH by sex (N = 3849)

The study found that the majority of both men and women had accepting attitudes towards PLWH (see Fig. 1). Over 90% believed that a female teacher with the AIDS virus but not sick should be allowed to continue teaching in school. More than 80% reported that they would be willing to care for a family member who became sick with the AIDS virus in their household. Additionally, almost 80% reported they would buy fresh vegetables from a shopkeeper they knew had the AIDS virus. Regarding the social stigma questions, 3% (n = 128) of older adults answered ‘no’ to all three stigma questions (scored 3 points); 8% (n = 308) answered ‘no’ to two of the questions (scored 2 points); 14% (n = 536) answered ‘no’ to one of the questions (scored 1 point); and 75% (n = 2877) answered ‘yes’ to all three questions (scored 0 points). For the anticipated stigma question, only a small proportion of both men (14.9%) and women (14.7%) reported that they would not want it to remain a secret if a family member became infected with the AIDS virus.

**Fig. 2:**
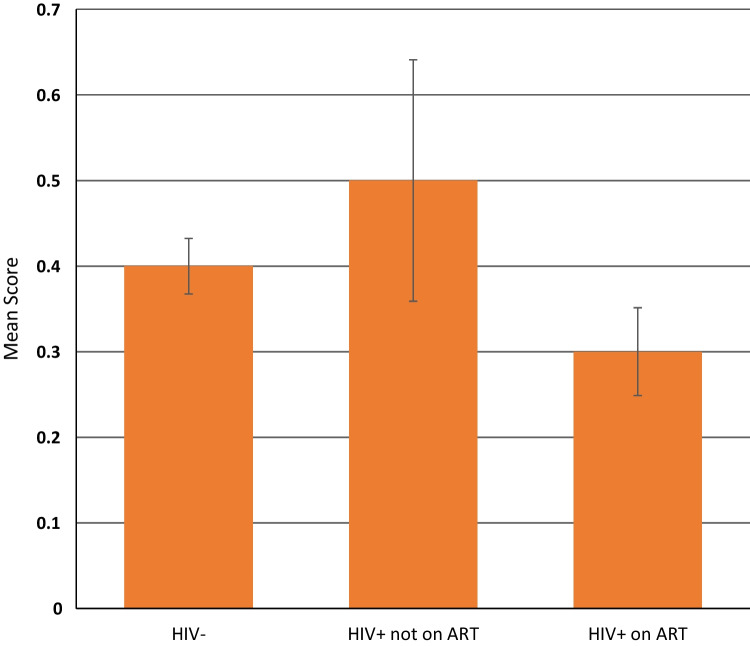
Social stigma by biological HIV and ART uptake status (N = 2931)

The study found that women significantly tend to have less social stigma compared to men (mean = 0.38 vs 0.42, t = 1.694, p = 0.045) (see Table 2). Younger respondents (mean = 0.26, F = 27.57, p = 0.000), those with secondary or more education (mean = 0.28, F = 14.46, p = 0.000), currently married or living with a partner (mean = 0.33, F = 8.35, p = 0.000), employed (mean = 0.27, F = 13.41, p = 0.000), living in 7+ person household (mean = 0.37, F = 3.35, p = 0.018), living in households with higher wealth asset index (mean = 0.33, F = 6.62, p = 0.000), those ever tested for HIV (mean = 0.35, t = − 7.684, p = 0.000), and those who tested HIV+ (mean = 0.29, F = 32.23, p = 0.000) significantly had less social stigma compared to older respondents (mean = 0.70, F = 27.57, p = 0.000), those with no formal education (mean = 0.45, F = 14.46, p = 0.000), never married (mean = 0.47, F = 8.35, p = 0.000), widowed (mean = 0.46, F = 8.35, p = 0.000), separated or divorced (mean = 0.43, F = 8.35, p = 0.000), unemployed (mean = 0.41, F = 13.41, p = 0.000), retired (mean = 0.47, F = 13.41, p = 0.000), living alone (mean = 0.50, F = 3.35, p = 0.018), in households with lower wealth asset index (mean = 0.50, F = 6.62, p = 0.000), never tested for HIV (mean = 0.57, t = − 7.684, p = 0.000), and those who tested HIV negative (mean = 0.40, F = 32.23, p = 0.000). There were no significant differences in anticipated stigma based on respondent’s covariates including sex, age group, marital status, education, employment, household size, household wealth asset index, ever tested for HIV and HIV status (see Table 3). However, there were significant differences in social stigma based on respondents’ biological HIV and ART uptake status (see Fig. 2). Older adults HIV+ on ART were significantly less likely to have social stigma compared to those HIV negative or HIV+ not on ART.

**Table 2:** Variations in social stigma score by respondents’ socio-demographics (N = 3849)

Variables	Mean (SD)		p value
Sex		t = 1.694	0.045
Male	0.42 (0.80)		
Female	0.38 (0.76)		
Age group		F = 27.57	0.000
40–49	0.26 (0.64)		
50–59	0.32 (0.70)		
60–69	0.35 (0.71)		
70–79	0.46 (0.82)		
80+	0.70 (0.98)		
Education		F = 14.46	0.000
No formal	0.45 (0.83)		
At least some primary (1–7 years)	0.40 (0.77)		
Secondary or more (8+ years)	0.28 (0.63)		
Marital status		F = 8.35	0.000
Never married	0.47 (0.82)		
Separated or divorced	0.43 (0.81)		
Widowed	0.46 (0.83)		
Currently married or living with partner	0.33 (0.71)		
Employment status		F = 13.41	0.000
Unemployed	0.41 (0.78)		
Employed (full-time or part-time)	0.27 (0.64)		
Retired	0.47 (0.83)		
Household size		F = 3.35	0.018
Living alone	0.50 (0.91)		
Living with another person	0.42 (0.81)		
Living in 3–6 person	0.39 (0.75)		
Living in 7+ person	0.37 (0.74)		
Wave 2 wealth asset index		F = 6.62	0.000
Index 1 (lower)	0.50 (0.86)		
Index 2	0.39 (0.76)		
Index 3	0.40 (0.79)		
Index 4	0.38 (0.76)		
Index 5 (higher)	0.33 (0.70)		
Wave 2 ever tested for HIV (self-report)		t = − 7.684	0.000
Yes	0.35 (0.73)		
No	0.57 (0.89)		
Wave 2 DBS HIV test result		F = 32.23	0.000
HIV+	0.29 (0.68)		
HIV−	0.40 (0.78)

**Table 3:** Anticipated stigma by socio-demographic characteristics (N = 3849)

Variables	% Saying will keep family member HIV status secret	Chi-square (χ^2^)	p value
Sex		χ^2^ (1) = 0.010	0.920
Male	85.17		
Female	85.29		
Age group		χ^2^ (4) = 0.448	0.978
40–49	85.29		
50–59	84.79		
60–69	85.75		
70–79	85.28		
80+	84.87		
Education		χ^2^ (2) = 2.600	0.273
No formal	86.29		
At least some primary (1–7 years)	84.52		
Secondary or more (8+ years)	84.29		
Marital status		χ^2^ (3) = 6.036	0.110
Never married	82.01		
Separated or divorced	83.37		
Widowed	84.88		
Currently married or living with partner	86.51		
Employment status		χ^2^ (2) = 1.936	0.380
Unemployed	84.58		
Employed (full-time or part-time)	86.24		
Retired	86.17		
Household size		χ^2^ (3) = 2.149	0.542
Living alone	83.41		
Living with another person	84.96		
Living in 3–6 person	85.03		
Living in 7+ person	86.17		
Wave 2 wealth asset index		χ^2^ (4) = 6.623	0.157
Index 1 (lower)	84.11		
Index 2	86.26		
Index 3	88.98		
Index 4	86.45		
Index 5 (higher)	83.90		
Wave 2 ever tested for HIV (self-report)		χ^2^ (1) = 2.552	0.110
Yes	86.44		
No	84.85		
Wave 2 DBS HIV test result		χ^2^ (2) = 0.304	0.859
HIV+	86.01		
HIV−	86.05

In the multivariable logistic regression analysis, social stigma was significantly associated with lower testing rates (OR = 0.66, 95% CI 0.53–0.81, p = 0.000), (OR = 0.61, 95% CI 0.47–0.79, p = 0.000), (OR = 0.56, 95% CI 0.38–0.83, p = 0.004) (see Table 4). No significant associations in testing were observed for sex and employment status. Age was a significant factor affecting testing, with older age groups significantly showing lower testing rates (OR = 0.46, 95% CI 0.32–0.65, p = 0.000), (OR = 0.33, 95% CI 0.23–0.48, p = 0.000) compared to younger age groups. Marital status and educational attainment also played a significant role in testing rates, with higher testing observed among those who were currently married (OR = 2.58, 95% CI 1.92–3.47, p = 0.000), separated or divorced (OR = 2.13, 95% CI 1.50–3.06, p = 0.000), widowed (OR = 2.07, 95% CI 1.52–2.83, p = 0.000), and those with some primary (OR = 1.22, 95% CI 1.02–1.47, p = 0.032), and secondary or more education (OR = 1.32, 95% CI 1.02–1.72, p = 0.035). Household size was only significantly associated with lower HIV testing among those living in households with 7 + people (OR = 0.71, 95% CI 0.53–0.95, p = 0.021). Conversely, HIV testing rates were higher among those in the wealth asset index 2 (OR = 1.28, 95% CI 1.03–1.58, p = 0.027) and higher (OR = 1.38, 95% CI 1.08–1.74, p = 0.008) compared to the lower wealth asset index category.

**Table 4:** Association between social stigma and self-report HIV testing among older adults (N = 3837)

Ever tested	Odd ratio	95% Conf. interval	p value
Social stigma score			
0	REF	REF	REF
1	0.66	(0.53–0.81)	0.000
2	0.61	(0.47–0.79)	0.000
3	0.56	(0.38–0.83)	0.004
Sex			
Male	REF	REF	REF
Female	1.08	(0.90–1.30)	0.416
Age group			
40–49	REF	REF	REF
50–59	0.78	(0.57–1.07)	0.116
60–69	0.73	(0.53–1.02)	0.067
70–79	0.46	(0.32–0.65)	0.000
80+	0.33	(0.23–0.48)	0.000
Marital status			
Never married	REF	REF	REF
Separated or divorced	2.13	(1.50–3.06)	0.000
Widowed	2.07	(1.52–2.83)	0.000
Currently married or living with partner	2.58	(1.92–3.47)	0.000
Education			
No formal	REF	REF	REF
Some primary (1–7 years)	1.22	(1.02–1.47)	0.032
Secondary or more (8+ years)	1.32	(1.02–1.72)	0.035
Employment			
Unemployed	REF	REF	REF
Employed (part or full time)	1.07	(0.83–1.37)	0.592
Retired	0.89	(0.74–1.08)	0.249
Household Size			
Living alone	REF	REF	REF
Living with another person	0.92	(0.66–1.29)	0.664
Living in 3–6-person household	1.02	(0.77–1.35)	0.894
Living in 7+ person household	0.71	(0.53–0.95)	0.021
Wave 2 wealth asset index			
Index 1 (lower)	REF	REF	REF
Index 2	1.28	(1.03–1.58)	0.027
Index 3	0.89	(0.65–1.27)	0.490
Index 4	1.07	(0.82–1.38)	0.613
Index 5 (higher)	1.38	(1.08–1.74)	0.008

Anticipated stigma was associated with higher HIV testing, although this association was not statistically significant (see Table 5). Age remained a significant factor, with significantly lower testing rates observed among those in older age groups (OR = 0.71, 95% CI 0.51–0.99, p = 0.044), (OR = 0.44, 95% CI 0.31–0.62, p = 0.000), (OR = 0.30, 95% CI 0.20–0.43, p = 0.000). Marital status played a significant role in HIV testing rates, with significantly higher testing rates observed among those currently married (OR = 2.68, 95% CI 1.99–3.59, p = 0.000), separated or divorced (OR = 2.14 95% CI 1.51–3.04, p = 0.000), widowed (OR = 2.10, 95% CI 1.54–2.87, p = 0.000) compared to those never married. Additionally, HIV testing was higher among individuals with some primary education (OR = 1.22, 95% CI 1.01–1.46, p = 0.034),  secondary or more education (OR = 1.34, 95% CI 1.03–1.73, p = 0.029), and those in wealth asset index 2 (OR = 1.30, 95% CI 1.05–1.61, p = 0.017) and higher wealth asset index (OR = 1.42, 95% CI 1.12–1.80, p = 0.004) compared to those with no formal education and those in lower wealth asset index category.

**Table 5:** Association between anticipated stigma and self-report HIV testing among older adults (N = 3837)

Ever tested	Odds ratio	95% Conf. interval	p value
0	REF	REF	REF
1. Anticipated stigma	1.19	(0.96–1.47)	0.106
Sex			
Male	REF	REF	REF
Female	1.09	(0.92–1.31)	0.321
Age			
40–49	REF	REF	REF
50–59	0.76	(0.56–1.05)	0.094
60–69	0.71	(0.51–0.99)	0.044
70–79	0.44	(0.31–0.62)	0.000
80+	0.30	(0.20–0.43)	0.000
Marital status			
Never married	REF	REF	REF
Separated or divorced	2.14	(1.51–3.04)	0.000
Widowed	2.10	(1.54–2.87)	0.000
Currently married or living with partner	2.68	(1.99–3.59)	0.000
Education			
No formal	REF	REF	REF
Some primary (1–7 years)	1.22	(1.01–1.46)	0.034
Secondary or more (8 + years)	1.34	(1.03–1.73)	0.029
Employment			
Unemployed	REF	REF	REF
Employed (part or full time)	1.08	(0.84–1.39)	0.534
Retired	0.89	(0.74–1.08)	0.253
Household size			
Living alone	REF	REF	REF
Living with one other person	0.92	(0.66–1.29)	0.630
Living in 3–6 person household	1.01	(0.76–1.32)	0.971
Living in 7+ person household	0.70	(0.53–0.93)	0.016
Wave 2 wealth asset index			
Index 1 (lower)	REF	REF	REF
Index2	1.30	(1.05–1.61)	0.017
Index3	0.90	(0.64–1.26)	0.545
Index4	1.09	(0.84–1.42)	0.519
Index 5 (higher)	1.42	(1.12–1.80)	0.004

In the multivariable logistic regression analysis, social stigma was significantly associated with lower ART uptake, particularly among those with a stigma score of 2 (OR = 0.41, 95% CI 0.19–0.87, p = 0.020) (see Table 6). No significant associations were found between ART uptake and sex, age group, education, and household size. However, significant associations were observed for marital status, with higher ART uptake among those widowed (OR = 2.73, 95% CI 1.33–5.61, p = 0.006) and currently married or living with a partner (OR = 2.21, 95% CI 1.09–4.50, p = 0.028) compared to those never married. ART uptake was also significantly higher among individuals in wealth asset index 3 (OR = 3.45, 95% CI 1.13–10.50, p = 0.029) compared to those in lower wealth asset index. On the other hand, individuals who were retired had a significantly lower ART uptake (OR = 0.54, 95% CI 0.29–1.01, p = 0.054). For anticipated stigma, no significant associations were observed for ART uptake based on sex, age, education, and employment (see Table 7). However, ART uptake was significantly higher among those widowed (OR = 2.92, 95% CI 1.42–5.98, p = 0.003), currently married or living with a partner (OR = 2.39, 95% CI 1.18–4.83, p = 0.016), and those in wealth asset index 3 (OR = 3.49, 95% CI 1.15–10.57, p = 0.027). Conversely, ART uptake was significantly lower among those living in households with 7 + people (OR = 0.49, 95% CI 0.25–0.99, p = 0.047).

**Table 6:** Association between social stigma and ART uptake among older adults living with HIV (N = 715)

ART uptake	Odds ratio	95% Conf. interval	p value
Social stigma score			
0	REF	REF	REF
1	0.83	(0.45–1.53)	0.556
2	0.41	(0.19–0.87)	0.020
3	0.44	(0.15–1.23)	0.125
Sex			
Male	REF	REF	REF
Female	1.03	(0.65–1.63)	0.894
Age group			
40–49	REF	REF	REF
50–59	0.97	(0.57–1.64)	0.898
60–69	1.39	(0.76–2.53)	0.283
70–79	1.39	(0.59–3.28)	0.455
80+	2.71	(0.55–13.34)	0.221
Marital status			
Never married	REF	REF	REF
Separated or divorced	1.15	(0.57–2.32)	0.706
Widowed	2.73	(1.33–5.61)	0.006
Currently married or living with partner	2.21	(1.09–4.50)	0.028
Education			
No formal	REF	REF	REF
Some primary (1–7 years)	0.85	(0.53–1.37)	0.511
Secondary or more (8+ years)	1.02	(0.57–1.81)	0.951
Employment			
Unemployed	REF	REF	REF
Employed (part or full time)	0.66	(0.40–1.07)	0.090
Retired	0.54	(0.29–1.01)	0.054
Household size			
Living alone	REF	REF	REF
Living with another person	1.22	(0.51–2.94)	0.654
Living in 3–6 person	0.85	(0.44–1.64)	0.631
Living in 7+ person	0.53	(0.26–1.06)	0.071
Wave 2 wealth asset index			
Index 1 (lower)	REF	REF	REF
Index 2	1.27	(0.75–2.16)	0.374
Index 3	3.45	(1.13–10.50)	0.029
Index 4	1.18	(0.59–2.35)	0.634
Index 5 (higher)	1.22	(0.68–2.18)	0.510

**Table 7:** Association between anticipated stigma and ART uptake among older adults living with HIV (N = 715)

ART uptake	Odds ratio	95% Conf. interval	p value
0	REF	REF	REF
1. Anticipated stigma	1.50	(0.89–2.54)	0.129
Sex			
Male	REF	REF	REF
Female	1.09	0.70–1.71)	0.694
Age			
40–49	REF	REF	REF
50–59	0.96	(0.57–1.63)	0.885
60–69	1.35	(0.74–2.47)	0.323
70–79	1.31	(0.55–3.09)	0.542
80+	2.58	(0.53–12.64)	0.241
Marital status			
Never married	REF	REF	REF
Separated or divorced	1.18	(0.59–2.38)	0.636
Widowed	2.92	(1.42–5.98)	0.003
Currently married or living with partner	2.39	(1.18–4.83)	0.016
Education			
No formal	REF	REF	REF
Some primary (1–7 years)	0.82	(0.51–1.32)	0.421
Secondary or more (8+ years)	0.98	(0.55–1.75)	0.959
Employment			
Unemployed	REF	REF	REF
Employed (part or full time)	0.70	(0.43–1.14)	0.149
Retired	0.56	(0.30–1.03)	0.061
Household size			
Living alone	REF	REF	REF
Living with one other person	1.23	(0.51–2.96)	0.639
Living in 3–6 person household	0.84	(0.44–1.61)	0.592
Living in 7+ person household	0.49	(0.25–0.99)	0.047
Wave 2 wealth asset index			
Index 1 (lower)	REF	REF	REF
Index 2	1.29	(0.76–2.18)	0.344
Index 3	3.49	(1.15–10.57)	0.027
Index 4	1.28	(0.65–2.53)	0.479
Index 5 (higher)	1.20	(0.67–2.15)	0.538

When analyzing data by age groups, the study found that social stigma was significantly associated with lower HIV testing rates among those with a stigma score of 1 in the age groups 50–59 (OR = 0.41, 95% CI 0.25–0.66, p = 0.000) and 70–79 (OR = 0.52, 95% CI 0.34–0.80, p = 0.003), and those with a score of 2 in the age groups 50–59 (OR = 0.53, 95% CI 0.30–0.96, p = 0.036) (see Table A1). HIV testing was significantly higher among females in the age groups 40–49 (OR = 1.93, 95% CI 1.05–3.55, p = 0.035) and 60–69 (OR = 1.46, 95% CI 1.02–2.08, p = 0.039), but significantly lower in the 80 + age group (OR = 0.60, 95% CI 0.36–1.00, p = 0.051). Those currently married, separated or divorced, and widowed had higher testing rates in the age groups 40–49 (OR = 2.17, 95% CI 1.07–4.40, p = 0.031), 50–59 (OR = 2.37, 95% CI 1.29–4.34, p = 0.005, (OR = 2.68, 95% CI 1.58–4.57, p = 0.000), (OR = 2.91, 95% CI 1.60–5.28, p = 0.000), 60–69 (OR = 1.97, 95% CI 1.02–3.79, p = 0.042), and 70–79 (OR = 4.69, 95% CI 1.78–12.34, p = 0.002), (OR = 2.71, 95% CI 1.26–5.85, p = 0.011), (OR = 5.31, 95% CI 2.45–11.49, p = 0.000). Education also had an impact on testing rates, with those with formal education showing higher testing rates in the age groups 50–59 (OR = 1.53, 95% CI 1.02–2.30, p = 0.042), (OR = 1.74, 95% CI 1.10–2.77, p = 0.019) and 60–69 (OR = 2.32, 95% CI 1.28–4.18, p = 0.005). Employment status did not show significant associations with HIV testing across all age groups. Household size was only significantly associated with lower testing rates among those living in 7 + person household in the 60–69 age group (OR = 0.45, 95% CI 0.24–0.82, p = 0.010). Wealth index was significantly associated with higher testing rates for those in wealth index 2 in the 40–49 age group (OR = 2.27, 95% CI 1.06–4.86, p = 0.035).

There was no significant association between anticipated stigma and HIV testing rates across all age groups (see Table A2). HIV testing was significantly higher among females in the age groups 40–49 (OR = 1.97, 95% CI 1.07–3.63, p = 0.029) and 60–69 (OR = 1.46, 95% CI 1.03–2.09, p = 0.036), but a significantly lower in the 80 + age group (OR = 0.59, 95% CI 0.36–0.98, p = 0.040). Those ever married had significantly higher testing rates in the age groups 40–49 (OR = 2.17, 95% CI 1.07–4.38, p = 0.031), 50–59 (OR = 2.23, 95% CI 1.23–4.04, p = 0.009), (OR = 2.72, 95% CI 1.61–4.60, p = 0.000), 60–69 (OR = 2.10, 95% CI 1.10–4.12, p = 0.025), and 70–79 (OR = 4.72, 95% CI 1.80–12.36, p = 0.002), (OR = 5.44, 95% CI 2.53–11.69, p = 0.000), while those widowed had significantly higher testing rates in the age groups 50–59 (OR = 2.90, 95% CI 1.61–5.24, p = 0.000) and 70–79 (OR = 2.75, 95% CI 1.29–5.87, p = 0.009). Those with more formal education showed significantly higher testing rates in the age groups 50–59 (OR = 1.65, 95% CI 1.04–2.62, p = 0.032) and 60–69 (OR = 2.29, 95% CI 1.27–4.12, p = 0.006). Employment status did not show significant associations with HIV testing across all age groups, except for lower testing rates among those retired in the age group 40–49 (OR = 0.24, 95% CI 0.06–0.99, p = 0.048). Household size was significantly associated with lower testing rates among those in 7 + person households in the age group 60–69 (OR = 0.44, 95% CI 0.24–0.80, p = 0.007), and higher testing rates were observed for those in wealth index 2 in the age group 40–49 (OR = 2.34, 95% CI 1.10–5.01, p = 0.028).

Social stigma was significantly associated with lower ART uptake among those with a social stigma score of 2 in the age groups 60–69 (OR = 0.20, 95% CI 0.04–0.91, p = 0.037) and 70–79 (OR = 0.01, 95% CI 0.00–1.03, p = 0.051) (see Table A3). No significant associations in ART uptake were observed for sex, marital status, education, and wealth index across all age groups. Employment was significantly associated with lower ART uptake only among those retired in the age group 60–69 (OR = 0.31, 95% CI 0.11–0.81, p = 0.017). Household size was significantly associated with lower ART uptake for those in 7+ person household in the age group 60–69 (OR = 0.11, 95% CI 0.02–0.64, p = 0.014). Anticipated stigma was significantly associated with higher ART uptake among those in the age group 60–69 (OR = 3.43, 95% CI 1.29–9.11, p = 0.013) (see Table A4). Marital status was significantly associated with higher ART uptake among those widowed in the age group 50–59 (OR = 4.14, 95% CI 1.05–16.23, p = 0.042). Employment was significantly associated with lower ART uptake among those retired in the age group 60–69 (OR = 0.36, 95% CI 0.14–0.93, p = 0.035). A significantly lower ART uptake was observed for those living in 7 + person households in the age group 70–79 (OR = 0.11, 95% CI 0.02–0.58, p = 0.009).

Social stigma was significantly associated with lower HIV testing among both men and women with a stigma score of 1(OR = 0.58, 95% CI 0.42–0.79, p = 0.001), (OR = 0.71, 95% CI 0.53–0.96, p = 0.026), score of 2 (OR = 0.64, 95% CI 0.43–0.96, p = 0.029), (OR = 0.58, 95% CI 0.41–0.83, p = 0.003) and score of 3 for men (OR = 0.44, 95% CI 0.26–0.76, p = 0.003) (see Table A5). Age group was significantly associated with lower testing among women in all age groups (OR = 0.55, 95% CI 0.35–0.87, p = 0.011), (OR = 0.59, 95% CI 0.36–0.95, p = 0.032), (OR = 0.30, 95% CI 0.18–0.51, p = 0.000), (OR = 0.21, 95% CI 0.12–0.37, p = 0.000) and for men only significantly associated with lower testing in 80 + age group (OR = 0.45, 95% CI 0.26–0.76, p = 0.003). Marital status was significantly associated with higher testing among both men and women, for those ever married (OR = 1.74, 95% CI 1.07–2.82, p = 0.026), (OR = 2.18, 95% CI 1.28–3.69, p = 0.004), (OR = 2.99, 95% CI 2.02–4.44, p = 0.000), (OR = 1.99, 95% CI 1.25–3.17, p = 0.004) and widowed (OR = 2.09, 95% CI 1.31–3.33, p = 0.002), (OR = 01.87, 95% CI 1.18–2.95, p = 0.008). Household size was significantly associated with less testing for men compared to women (OR = 0.51, 95% CI 0.32–0.81, p = 0.004), (OR = 0.65, 95% CI 0.43–0.98, p = 0.039), (OR = 0.53, 95% CI 0.34–0.82, p = 0.004), and significantly higher for men in the highest wealth index (OR = 1.65, 95% CI 1.14–2.38, p = 0.007) and significantly higher for women in the second wealth index (OR = 1.38, 95% CI 1.03–1.85, p = 0.033).

Anticipated stigma was significantly associated with higher HIV testing for both men and women, although this association was not significant (see Table A6). Age group was significantly associated with lower testing among women in all age groups (OR = 0.54, 95% CI 0.34–0.86, p = 0.009), (OR = 0.56, 95% CI 0.35–0.92, p = 0.021), (OR = 0.29, 95% CI 0.17–0.48, p = 0.000), (OR = 0.19, 95% CI 0.11–0.32, p = 0.000), and significantly lower for men only in the 70–79 (OR = 0.59, 95% CI 0.36–0.97, p = 0.038) and 80 + age group (OR = 0.41, 95% CI 0.24–0.70, p = 0.001). Marital status was significantly associated with higher testing for both men and women, for currently married (OR = 3.06, 95% CI 2.07–4.53, p = 0.000), (OR = 2.07, 95% CI 1.30–3.29, p = 0.002), widowed (OR = 2.11, 95% CI 1.33–3.35, p = 0.001), (OR = 1.92, 95% CI 1.21–3.02, p = 0.005), and separated or divorced (OR = 1.76, 95% CI 1.09–2.85, p = 0.021), (OR = 2.19, 95% CI 1.29–3.71, p = 0.004). Household size was significantly associated with less testing for men compared to women (OR = 0.53, 95% CI 0.34–0.85, p = 0.007), (OR = 0.67, 95% CI 0.45–1.01, p = 0.053), (OR = 0.54, 95% CI 0.35–0.84, p = 0.006). Wealth index was significantly associated with higher testing among men in the highest wealth index (OR = 1.71, 95% CI 1.19–2.46, p = 0.004) and for women in the second wealth index (OR = 1.41, 95% CI 1.05–1.89, p = 0.022).

Social stigma was significantly associated with lower ART uptake for men with a stigma score of 3 (OR = 0.28, 95% CI 0.08–0.97, p = 0.046) and women with a score of 2 (OR = 0.28, 95% CI 0.10–0.83, p = 0.022) (see Table A7). ART uptake was significantly higher among men in the age group 60–69 compared to women (OR = 2.61, 95% CI 1.01–6.78, p = 0.048). Being widowed was significantly associated with higher ART uptake among men (OR = 5.90, 95% CI 1.45–24.00, p = 0.013). Employment was significantly associated with lower ART uptake among women compared to men (OR = 0.34, 95% CI 0.18–0.68, p = 0.002), while living in a 7 + person household was significantly associated with lower ART uptake among men (OR = 0.30, 95% CI 0.10–0.90, p = 0.032). Anticipated stigma was significantly associated with higher ART uptake among men compared to women (OR = 2.35, 95% CI 1.07–5.14, p = 0.033) (see Table A8), with age group 60–69 also showing significantly higher ART uptake for men compared to women (OR = 2.61, 95% CI 1.00–6.79, p = 0.049). Being widowed and currently married or living with a partner was significantly associated with higher ART uptake among men compared to women (OR = 6.28, 95% CI 1.55–25.41, p = 0.010), (OR = 2.78, 95% CI 0.99–7.81, p = 0.053). Education and employment were significantly associated with lower ART uptake among women compared to men, in particular for those with some primary education and those employed (OR = 0.52, 95% CI 0.27–0.99, p = 0.048), (OR = 0.36, 95% CI 0.19–0.70, p = 0.003), and living in a 7 + person household was significantly associated with lower ART uptake among men compared to women (OR = 0.30, 95% CI 0.10–0.92, p = 0.035).

## Discussion

This study examined the association between HIV-related stigma and HIV testing and ART uptake in a cohort of older adults in rural South Africa. We found that higher social stigma scores were associated with a significant decrease in the likelihood of ever testing for HIV among all participants with social stigma. This finding suggests that social stigma poses a significant barrier to testing behaviour among older adults. HIV testing is an important gateway to accessing HIV prevention and care and treatment services. However, HIV-related stigma is widely recognized as a major obstacle to successful HIV control efforts. Previous research has shown that stigma not only affects HIV testing uptake, but also creates challenges for PLWH in accessing care, starting treatment, and adhering to ART [44–46]. Bessong et al. [47] asserts that limitations in access to HIV testing or treatment for PLWH due to stigma could affect the preventative impact of ART, making it essential to address HIV-related stigma to improve the effectiveness of HIV control initiatives.

Several studies have reported on the association between HIV-related stigma and uptake of HIV testing. Similar to the findings of this study, these studies have shown that stigma is a significant barrier to HIV testing [26–31, 48, 49]. Studies from different countries, such as Botswana and Venezuela, have also highlighted stigma as the primary obstacle to HIV testing [50, 51]. Furthermore, systematic reviews from India and other regions have also consistently identified HIV-related stigma as a key reason for low HIV testing uptake and a common barrier to linkage to HIV care and accessing ART services [46, 52–56], indicating that stigma influences every stage of the HIV care continuum. These findings collectively underscore the critical role of addressing HIV-related stigma to improve HIV testing uptake and care outcomes.

HIV-related stigma negatively influences HIV testing uptake in several ways. Stigma creates a fear of discrimination and negative social consequences for those perceived to have HIV. This fear may discourage people from getting tested as they may worry about being treated differently or ostracized if their HIV status becomes known. Findings from previous research [19, 30, 51, 53, 57] also suggest that fears surrounding the test and the possibility of a positive diagnosis, often due to stigma, also deter people from seeking HIV testing. HIV-related stigma can also lead to feelings of shame and guilt associated with HIV risk behaviors, and these feelings can discourage individuals engaging in risk behaviours from getting tested and treated for HIV as they may associate a positive result with personal failure of moral judgement. Stigma is also fueled by misunderstanding about HIV transmission [58]. Misconceptions about HIV transmission may lead some to believe they are not at risk and avoid testing. HIV-related stigma may also reduce social networks and social interactions for PLWH because of self-imposed social isolation and avoiding negative judgement and guilt related to HIV [39]. People may avoid testing as they fear losing social support or connections if diagnosed with HIV. Fear of disclosing HIV status to family, friends, or partners due to stigma is a significant barrier to testing. Studies revealed that the sharing of positive HIV status with family or friends may lead to social stigma [59] and isolation or exclusion by community [22, 60]. Therefore, individuals may choose to not get tested to avoid the potential consequences of having to disclose their status.

Cultural and religious beliefs can also contribute to HIV-related stigma, resulting in hesitancy in testing. In some communities, HIV/AIDS is blamed on witchcraft, spirits and supernatural forces [61], and still seen by some as a form of religious punishment for a culpable person, a curse from God or a sinner’s disease [62]. These stigmatizing beliefs can discourage individuals from seeking HIV testing and care, further perpetuating the negative impact of HIV-related stigma. Additionally, concerns about confidentiality of HIV tests results can contribute to lower uptake of HIV testing, consistent with [46, 57]. Individuals may fear that their HIV status will not be kept private, leading them to avoid testing altogether. HIV testing is the key entry point into the HIV care cascade, and without improvements in testing, it will be impossible to reach the UNAIDS 95–95–95 targets. Thus, addressing HIV-related stigma through awareness, education, and creating supportive environments is essential to encourage testing and increase the uptake of HIV testing among older adults. Furthermore, ensuring confidential, accessible and non-judgmental testing services can encourage individuals to get tested, and access care and support if needed.

The study found evidence supporting a negative association between HIV-related stigma and ART uptake among older adults living with HIV. Social stigma was significantly linked to lower levels of ART uptake, especially among those with a social stigma score of 2. This finding aligns with the results of two systematic reviews and meta-analyses studies, which also found significant correlations between HIV-related stigma and ART adherence [45, 63]. The studies indicated that HIV-related stigma negatively impacted adherence to ART by compromising general psychological processes, such as social support and adaptive coping. PLWH facing discrimination and rejection may experience reduced social support, which plays a crucial role in maintaining adherence. Previous research has also demonstrated the importance of social ties in promoting adherence, particularly in resource-limited settings [64]. HIV-related stigma can also hinder individuals’ ability to cope adaptively with the challenges of living with HIV, potentially affecting their medication adherence and overall well-being [45]. PLWH who experience enacted stigma and anticipated stigma may resort to concealing their status, leading to delays in treatment initiation and interruptions in treatment uptake. Stigma-related fears and concealment practices can disrupt continuity of care and medication adherence, posing challenges to effectively managing HIV. The study findings are also supported by other studies conducted in South Africa [65] and Tanzania [66] which also highlight how widespread HIV-related stigma affects HIV testing willingness and treatment adherence. ART uptake is crucial for improving the health outcomes of PLWH, but stigma can act as a barrier to its success. Addressing HIV-related stigma is essential, particularly for older adults, to enhance ART uptake and quality of life. Effective interventions that target HIV-related stigma are needed to improve ART uptake among older populations and promote better health outcomes.

In this study, there was a positive association between anticipated stigma and ART uptake, but it was not statistically significant. Previous research on the association between anticipated stigma and ART adherence has shown inconsistent results [24, 67]. Some studies found no association or a non-significant one [68, 69], while others observed both positive and negative associations [70, 71]. These inconsistencies may be due to variations in participants and measurement methods for anticipated stigma [24]. However, it is also possible that anticipated stigma's impact on ART adherence depends on other unmeasured psychosocial factors. For example, the study by [24] revealed that anticipated stigma was not significantly associated with ART non-adherence, but when accounting for medication concerns, anticipated stigma became associated with increased ART adherence. Future research on HIV-related stigma should use standardized measures of anticipated stigma and include prospective analyses to explore potential mediating variables.

The study also identified several key factors influencing HIV testing and ART uptake among older adults. Older age was associated with reduced HIV testing, consistent with other research showing lower testing rates among older individuals [72]. Lack of older adults HIV prevention programs, low risk perception, and stigma contribute to this trend [19, 57, 73]. The increasing number of PLWH aged 50 years and above highlights the need for integrating older adults into HIV prevention, care, and treatment programs to effectively address the HIV epidemic. Being ever married increased the likelihood of both HIV testing and ART uptake, consistent with other studies [74–81] and possibly due to perceived risk within relationships and premarital HIV counselling and testing. Consistent with other studies [75, 78, 82, 83], higher educational levels were positively associated with HIV testing, likely because education influences health awareness and access to testing services [84, 85]. Wealthier individuals also had higher odds of HIV testing and ART uptake, in line with other studies [77, 86–88]. Social stigma was found to be higher among older adults, in line with [89, 90]. HIV and ART status were significant predictors of stigma, and anticipated stigma was observed as a barrier to disclosing HIV status among older adults in the study.

## Strengths and Limitations

This study's strengths include its integration into an old age cohort with a large sample size from an HDSS platform, allowing for generalizability to the study area's population. The longitudinal cohort design contributes to low rates of loss to follow-up, and the use of DBS provides biological measurements for HIV status, viral load, and ART. However, there are limitations in the study. We used self-reporting for HIV testing which may introduce social desirability bias. The cross-sectional design prevents causal interpretations of associations. Other dimensions of HIV-related stigma beyond social and anticipated stigma were not assessed. The assessment of anticipated stigma was limited to one question, which may not fully capture its complexity. Additionally, the measures of HIV stigma rely on self-reports of hypothetical scenarios, which might lead to misconceptions by respondents.

## Conclusion

The study results indicate a significant negative influence of HIV-related stigma on HIV testing and ART uptake among older adults in rural South Africa. This emphasizes the need to address HIV-related stigma as a vital aspect on efforts to increase testing and treatment rates in this population. By implementing targeted interventions to combat stigma, we can make substantial progress towards achieving the UNAIDS 95–95–95 targets.

### Supplementary Information

Below is the link to the electronic supplementary material.

Supplementary file1 (DOCX 93 KB)
