# Supplementary material for: The Association Between HIV-Related Stigma and the Uptake of HIV Testing and ART Among Older Adults in Rural South Africa: Findings from the HAALSI Cohort Study
Source: AIDS Behav. 2024 Jan 29;28(3):1104–21. doi: 10.1007/s10461-023-04222-w (PMC10896802; doi:10.1007/s10461-023-04222-w)
Supplement: Supplementary file 1 — Supplementary file1 (DOCX 93 KB) [file 10461_2023_4222_MOESM1_ESM.docx]

Supplementary Material

Table of Contents

[**Appendix A: Supplementary Analyses** 2](#_Toc142206274)

[Table A1: Association between social stigma and self-report HIV testing among older adults by age group 2](#_Toc142206275)

[Table A2: Association between anticipated stigma and self-report HIV testing among older adults by age group..3](#_Toc142206276)

[Table A3: Association between social stigma and ART uptake among PLWH by age group 4](#_Toc142206277)

[Table A4: Association between anticipated stigma and ART uptake among PLWH by age group 5](#_Toc142206278)

[Table A5: Association between social stigma and self-report HIV testing among older adults by sex 6](#_Toc142206279)

[Table A6: Association between anticipated stigma and self-report HIV testing among older adults by sex 7](#_Toc142206280)

[Table A7: Association between social stigma and ART uptake among PLWH by sex 8](#_Toc142206281)

[Table A8: Association between anticipated stigma and ART uptake among PLWH by sex 9](#_Toc142206282)

#### **Appendix A: Supplementary Analyses**

#### **Table A1 Association between social stigma and self-report HIV testing among older adults by age group**

|  | **Age 40-49** | | **Age 50-59** | | **Age 60-69** | | **Age 70-79** | | **Age 80+** | |
| --- | --- | --- | --- | --- | --- | --- | --- | --- | --- | --- |
| **Ever tested** | **Odds ratio (95% CI)** | **P-value** | **Odds ratio (95% CI)** | **P-value** | **Odds ratio (95% CI)** | **P-value** | **Odds ratio (95% CI)** | **P-value** | **Odds ratio (95% CI)** | **P-value** |
| **Social Stigma Score** |  |  |  |  |  |  |  |  |  |  |
| 0 | REF | REF | REF | REF | REF | REF | REF | REF | REF | REF |
| 1 | 0.84 (0.40-1.91) | 0.679 | 0.41 (0.25-0.66) | 0.000*** | 0.71 (0.47-1.07) | 0.103 | 0.52 (0.34-0.80) | 0.003** | 1.15(0.70-1.90) | 0.585 |
| 2 | 0.58 (0.19-1.79) | 0.344 | 0.53 (0.30-0.96) | 0.036* | 0.59 (0.35-1.02) | 0.059 | 0.66 (0.39-1.12) | 0.126 | 0.62 (0.35-1.10) | 0.102 |
| 3 | 0.36 (0.08-1.60) | 0.178 | 0.55 (0.20-1.51) | 0.244 | 0.84 (0.32-2.18) | 0.714 | 0.59 (0.27-1.26) | 0.171 | 0.57 (0.29-1.14) | 0.114 |
| **Sex** |  |  |  |  |  |  |  |  |  |  |
| Male | REF | REF | REF | REF | REF | REF | REF | REF | REF | REF |
| Female | 1.93 (1.05-3.55) | 0.035* | 0.81 (0.55-1.18) | 0.265 | 1.46 (1.02-2.08) | 0.039* | 1.11 (0.76-1.64) | 0.585 | 0.60 (0.36-1.00) | 0.051* |
| **Marital Status** |  |  |  |  |  |  |  |  |  |  |
| Never Married | REF | REF | REF | REF | REF | REF | REF | REF | REF | REF |
| Separated or divorced | 1.54 (0.67-3.53) | 0.308 | 2.37 (1.29-4.34) | 0.005** | 1.39 (0.67-2.86) | 0.378 | 4.69 (1.78-12.34) | 0.002** | 1.57 (0.38-6.46) | 0.532 |
| Widowed | 1.03 (0.39-2.74) | 0.947 | 2.91 (1.60-5.28) | 0.000*** | 1.52 (0.78-2.96) | 0.222 | 2.71 (1.26-5.85) | 0.011* | 1.86 (0.56-6.16) | 0.308 |
| Currently Married or Living w/ Partner | 2.17 (1.07-4.40) | 0.031* | 2.68 (1.58-4.57) | 0.000*** | 1.97 (1.02-3.79) | 0.042* | 5.31 (2.45-11.49) | 0.000*** | 1.13 (0.34-3.68) | 0.845 |
| **Education** |  |  |  |  |  |  |  |  |  |  |
| No formal | REF | REF | REF | REF | REF | REF | REF | REF | REF | REF |
| Some primary (1-7 years) | 0.63 (0.25-1.54) | 0.309 | 1.53 (1.02-2.30) | 0.042* | 1.36 (0.98-1.90) | 0.068 | 1.22 (0.85-1.75) | 0.284 | 0.93 (0.58-1.48) | 0.756 |
| Secondary or more (8+ years) | 0.53 (0.22-1.28) | 0.160 | 1.74 (1.10-2.77) | 0.019* | 2.32 (1.28-4.18) | 0.005** | 0.88 (0.47-1.64) | 0.695 | 0.50 (0.14-1.79) | 0.286 |
| **Employment** |  |  |  |  |  |  |  |  |  |  |
| Unemployed | REF | REF | REF | REF | REF | REF | REF | REF | REF | REF |
| Employed (part or full time) | 1.18 (0.64-2.17) | 0.600 | 0.91 (0.62-1.35) | 0.653 | 1.03 (0.64-1.66) | 0.915 | 0.73 (0.31-1.76) | 0.486 | 1.48 (0.25-8.88) | 0.668 |
| Retired | 0.25 (0.06-1.08) | 0.063 | 0.42 (0.15-1.13) | 0.087 | 0.72 (0.51-1.02) | 0.061 | 1.09 (0.78-1.51) | 0.615 | 1.00 (0.68-1.46) | 0.994 |
| **Household Size** |  |  |  |  |  |  |  |  |  |  |
| Living alone | REF | REF | REF | REF | REF | REF | REF | REF | REF | REF |
| Living with one other person | 1.05 (0.30-3.67) | 0.943 | 0.87 (0.40-1.88) | 0.717 | 0.65 (0.33-1.31) | 0.229 | 0.90 (0.44-1.83) | 0.765 | 1.72 (0.86-3.46) | 0.128 |
| Living in 3-6 person household | 0.83 (0.33-2.10) | 0.691 | 1.03 (0.54-1.96) | 0.920 | 0.78 (0.43-1.41) | 0.403 | 1.09 (0.59-2.01) | 0.783 | 1.54 (0.86-2.76) | 0.149 |
| Living in 7+ person household | 0.56 (0.21-1.50) | 0.247 | 0.84 (0.43-1.64) | 0.612 | 0.45 (0.24-0.82) | 0.010* | 0.66 (0.35-1.24) | 0.196 | 1.31 (0.70-2.50) | 0.407 |
| **Wave 2 Wealth Index Asset** |  |  |  |  |  |  |  |  |  |  |
| Lower | REF | REF | REF | REF | REF | REF | REF | REF | REF | REF |
| Index=2 | 2.27 (1.06-4.86) | 0.035* | 1.17 (0.73-1.88) | 0.504 | 1.12 (0.73-1.73) | 0.601 | 1.25 (0.79-2.00) | 0.340 | 1.31 (0.81-2.11) | 0.270 |
| Index=3 | 1.29 (0.42-3.96) | 0.660 | 0.68 (0.34-1.38) | 0.289 | 1.22 (0.63-2.36) | 0.563 | 0.80 (0.39-1.65) | 0.541 | 0.84 (0.37-1.93) | 0.684 |
| Index=4 | 1.80 (0.71-4.57) | 0.218 | 0.93 (0.52-1.65) | 0.795 | 1.05 (0.63-1.75) | 0.860 | 1.34 (0.77-2.33) | 0.306 | 0.75 (0.40-1.40) | 0.365 |
| Higher | 1.92 (0.84-4.40) | 0.124 | 1.38 (0.81-2.35) | 0.235 | 1.42 (0.89-2.28) | 0.145 | 1.13 (0.70-1.82) | 0.624 | 1.78 (0.99-3.21) | 0.056 |

*p<0.05, **p<0.01, ***p<0.001

#### **Table A2 Association between anticipated stigma and self-report HIV testing among older adults by age group**

*p<0.05, **p<0.01, ***p<0.001

|  | **Age 40-49** | | **Age 50-59** | | **Age 60-69** | | **Age 70-79** | | **Age 80+** | |
| --- | --- | --- | --- | --- | --- | --- | --- | --- | --- | --- |
| **Ever tested** | **Odds ratio (95% CI)** | **P-value** | **Odds ratio (95% CI)** | **P-value** | **Odds ratio (95% CI)** | **P-value** | **Odds ratio (95% CI)** | **P-value** | **Odds ratio (95% CI)** | **P-value** |
| 0 | REF | REF | REF | REF | REF | REF | REF | REF | REF | REF |
| **1.Anticipated stigma** | 1.52 (0.75-3.09) | 0.247 | 1.10 (0.70-1.74) | 0.677 | 1.21 (0.79-1.83) | 0.384 | 1.23 (0.80-1.90) | 0.348 | 1.01 (0.60-1.71) | 0.956 |
| **Sex** |  |  |  |  |  |  |  |  |  |  |
| Male | REF | REF | REF | REF | REF | REF | REF | REF | REF | REF |
| Female | 1.97 (1.07-3.63) | 0.029* | 0.85 (0.59-1.23) | 0.396 | 1.46 (1.03-2.09) | 0.036* | 1.14 (0.77-1.67) | 0.513 | 0.59 (0.36-0.98) | 0.040* |
| **Marital status** |  |  |  |  |  |  |  |  |  |  |
| Never married | REF | REF | REF | REF | REF | REF | REF | REF | REF | REF |
| Separated or divorced | 1.5 (0.66-3.44) | 0.326 | 2.23 (1.23-4.04) | 0.009** | 1.45 (0.70-2.99) | 0.313 | 4.72 (1.80-12.36) | 0.002** | 1.69 (0.41-6.88) | 0.466 |
| Widowed | 0.99 (0.37-2.62) | 0.987 | 2.90 (1.61-5.24) | 0.000*** | 1.59 (0.82-3.10) | 0.171 | 2.75 (1.29-5.87) | 0.009** | 2.03 (0.62-6.67) | 0.243 |
| Currently Married or Living w/ Partner | 2.17 (1.07-4.38) | 0.031* | 2.72 (1.61-4.60) | 0.000*** | 2.10 (1.10-4.02) | 0.025* | 5.44 (2.53-11.69) | 0.000*** | 1.21 (0.37-3.92) | 0.749 |
| **Education** |  |  |  |  |  |  |  |  |  |  |
| No formal | REF | REF | REF | REF | REF | REF | REF | REF | REF | REF |
| Some primary (1-7 years) | 0.59 (0.24-1.44) | 0.246 | 1.43 (0.95-2.13) | 0.084 | 1.35 (0.97-1.88) | 0.073 | 1.26 (0.88-1.80) | 0.212 | 0.92 (0.58-1.47) | 0.732 |
| Secondary or more (8+ years) | 0.53 (0.22-1.28) | 0.158 | 1.65 (1.04-2.62) | 0.032* | 2.29 (1.27-4.12) | 0.006** | 0.93 (0.50-1.73) | 0.824 | 0.55 (0.15-1.94) | 0.348 |
| **Employment** |  |  |  |  |  |  |  |  |  |  |
| Unemployed | REF | REF | REF | REF | REF | REF | REF | REF | REF | REF |
| Employed (part or full time) | 1.16 (0.63-2.15) | 0.626 | 0.96 (0.66-1.42) | 0.855 | 1.04 (0.64-1.67) | 0.886 | 0.75 (0.31-1.78) | 0.510 | 1.80 (0.31-10.52) | 0.514 |
| Retired | 0.24 (0.06-0.99) | 0.048* | 0.41 (0.15-1.11) | 0.078 | 0.73 (0.52-1.02) | 0.066 | 1.09 (0.78-1.51) | 0.621 | 1.04 (0.72-1.52) | 0.818 |
| **Household size** |  |  |  |  |  |  |  |  |  |  |
| Living alone | REF | REF | REF | REF | REF | REF | REF | REF | REF | REF |
| Living with one other person | 1.07 (0.30-3.74) | 0.920 | 0.88 (0.41-1.89) | 0.752 | 0.65 (0.32-1.29) | 0.214 | 0.86 (0.42-1.75) | 0.673 | 1.71 (0.86-3.43) | 0.128 |
| Living in 3-6 person household | 0.82 (0.33-2.08) | 0.679 | 1.01 (0.53-1.90) | 0.979 | 0.75 (0.42-1.36) | 0.349 | 1.01 (0.55-1.86) | 0.972 | 1.68 (0.95-2.99) | 0.075 |
| Living in 7+ person household | 0.57 (0.21-1.52) | 0.263 | 0.84 (0.44-1.64) | 0.616 | 0.44 (0.24-0.80) | 0.007** | 0.63 (0.34-1.17) | 0.145 | 1.38 (0.73-2.61) | 0.322 |
| **Wave 2 wealth asset index** |  |  |  |  |  |  |  |  |  |  |
| Lower | REF | REF | REF | REF | REF | REF | REF | REF | REF | REF |
| Index 2 | 2.34 (1.10-5.01) | 0.028* | 1.24 (0.78-1.97) | 0.370 | 1.12 (0.73-1.72) | 0.599 | 1.30 (0.82-2.06) | 0.270 | 1.31 (0.82-2.10) | 0.266 |
| Index 3 | 1.29 (0.42-3.98) | 0.653 | 0.72 (0.36-1.44) | 0.348 | 1.26 (0.65-2.44) | 0.496 | 0.79 (0.38-1.63) | 0.527 | 0.81 (0.35-1.84) | 0.612 |
| Index 4 | 1.84 (0.73-4.66) | 0.198 | 0.98 (0.55-1.74) | 0.955 | 1.07 (0.64-1.80) | 0.784 | 1.33 (0.77-2.32) | 0.306 | 0.74 (0.39-1.38) | 0.337 |
| Higher | 2.00 (0.87- 4.59) | 0.101 | 1.48 (0.88-2.51) | 0.142 | 1.48 (0.92-2.37) | 0.103 | 1.12 (0.69-1.79) | 0.651 | 1.78 (0.99-3.21) | 0.053 |

#### **Table A3 Association between social stigma and ART uptake among PLWH by age group**

|  | **Age 40-49** | | **Age 50-59** | | **Age 60-69** | | **Age 70-79** | | **Age 80+** | |
| --- | --- | --- | --- | --- | --- | --- | --- | --- | --- | --- |
| **Ever tested** | **Odds ratio (95% CI)** | **P-value** | **Odds ratio (95% CI)** | **P-value** | **Odds ratio (95% CI)** | **P-value** | **Odds ratio (95% CI)** | **P-value** | **Odds ratio (95% CI)** | **P-value** |
| **Social Stigma Score** |  |  |  |  |  |  |  |  |  |  |
| 0 | REF | REF | REF | REF | REF | REF | REF | REF | REF | REF |
| 1 | 0.56 (0.17-1.88) | 0.352 | 1.49 (0.45-4.95) | 0.512 | 1.99 (0.37-10.79) | 0.423 | 0.078 (0.00-2.84) | 0.164 |  |  |
| 2 | 1.83 (0.20-17.06) | 0.596 | 0.33 (0.084-1.29) | 0.112 | 0.20 (0.04-0.91) | 0.037* | 0.01 (0.00-1.03) | 0.051* |  |  |
| 3 | 0.62 (0.36-10.91) | 0.747 | 1.74 (0.16-19.28) | 0.653 | 0.25 (0.04-1.41) | 0.117 | 1 (empty) |  |  |  |
| **Sex** |  |  |  |  |  |  |  |  |  |  |
| Male | REF | REF | REF | REF | REF | REF | REF | REF | REF | REF |
| Female | 1.52 (0.59-3.93) | 0.387 | 1.12 (0.51-2.48) | 0.779 | 0.95 (0.35-2.59) | 0.917 | 3.27 (0.06-170.19) | 0.556 |  |  |
| **Marital Status** |  |  |  |  |  |  |  |  |  |  |
| Never Married | REF | REF | REF | REF | REF | REF | REF | REF | REF | REF |
| Separated or divorced | 2.10 (0.61-7.20) | 0.240 | 0.74 (0.20-2.72) | 0.649 | 0.97 (0.08-12.12) | 0.979 | 0.02 (0.00-19.45) | 0.271 |  |  |
| Widowed | 2.19 (0.56-8.60) | 0.260 | 3.51 (0.86-14.25) | 0.079 | 1.70 (0.13-21.67) | 0.681 | 2.57 (0.04-183.44) | 0.665 |  |  |
| Currently Married or Living w/ Partner | 1.74 (0.56-5.43) | 0.337 | 1.36 (0.34-5.48) | 0.665 | 3.79 (0.30-48.16) | 0.305 | 1.90 (0.03-135.89) | 0.767 |  |  |
| **Education** |  |  |  |  |  |  |  |  |  |  |
| No formal | REF | REF | REF | REF | REF | REF | REF | REF | REF | REF |
| Some primary (1-7 years) | 1.32 (0.38-4.59) | 0.657 | 0.55 (0.23-1.29) | 0.166 | 1.09 (0.44-2.70) | 0.859 | 0.94 (0.05-18.34) | 0.969 |  |  |
| Secondary or more (8+ years) | 1.20 (0.34-4.27) | 0.781 | 0.86 (0.32-2.30) | 0.764 | 1.71 (0.41-7.15) | 0.462 | 1 (empty) |  |  |  |
| **Employment** |  |  |  |  |  |  |  |  |  |  |
| Unemployed | REF | REF | REF | REF | REF | REF | REF | REF | REF | REF |
| Employed (part or full time) | 0.60 (0.24-1.47) | 0.262 | 0.52 (0.23-1.15) | 0.104 | 1.10 (0.27-4.55) | 0.897 | 0.02 (0.00-1.15) | 0.058 |  |  |
| Retired | 0.89 (0.13-6.24) | 0.904 | 0.89 (0.11-7.39) | 0.912 | 0.31 (0.11-0.81) | 0.017* | 1.29 (0.07-22.20) | 0.862 |  |  |
| **Household Size** |  |  |  |  |  |  |  |  |  |  |
| Living alone | REF | REF | REF | REF | REF | REF | REF | REF | REF | REF |
| Living with one other person | 2.62 (0.45-15.28) | 0.284 | 1.99 (0.42-9.44) | 0.387 | 0.24 (0.04-1.44) | 0.119 | 1 (empty) |  |  |  |
| Living in 3-6 person household | 1.57 (0.42-5.84) | 0.500 | 1.39 (0.48-4.05) | 0.544 | 0.31 (0.06-1.60) | 0.162 | 16.26 (0.57-461.99) | 0.102 |  |  |
| Living in 7+ person household | 0.84 (0.21-3.43) | 0.813 | 1.19 (0.36-3.92) | 0.772 | 0.11 (0.02-0.64) | 0.014* | 1 (empty) |  |  |  |
| **Wave 2 Wealth Index Asset** |  |  |  |  |  |  |  |  |  |  |
| Lower | REF | REF | REF | REF | REF | REF | REF | REF | REF | REF |
| Index=2 | 0.97 (0.33-2.88) | 0.955 | 1.79 (0.70-4.56) | 0.222 | 2.07 (0.63-6.78) | 0.231 | 0.02 (0.00-2.25) | 0.101 |  |  |
| Index=3 | 2.18 (0.21-22.60) | 0.514 | 2.67 (0.49-14.65) | 0.257 | 8.64 (0.83-90.43) | 0.072 | 1 (empty) |  |  |  |
| Index=4 | 0.85 (0.20-3.56) | 0.824 | 1.31 (0.42-4.12) | 0.646 | 1.74 (0.44-6.83) | 0.431 | 1 (empty) |  |  |  |
| Higher | 4.16 (0.83-20.74) | 0.082 | 1.52 (0.57-4.04)  *p<0.05. For age group70-79, several variables were found to have perfect prediction or collinearity issues, resulting in their omission from the analysis. The variable w2c_hhsize with a value of 0 predicts success perfectly, and with a value of 1 or 3, it predicts success perfectly as well. These cases were omitted, and the outcome data for a total of 6 observations (6 for each value) were not used. The variable w2c_wealthindex with a value of 3 or 4 predicts success perfectly. These cases were omitted, and the outcome data for a total of 5 observations (5 for value 3) and 6 observations (6 for value 4) were not used. The variable socialstigmascore_1 with a value of 3 predicts failure perfectly. This case was omitted, and the outcome data for 1 observation was not used. The variable w1c_bd_educ4 with a value of 3 predicts failure perfectly. This case was omitted, and the outcome data for 1 observation was not used. The variable w2c_hhsize was omitted from the analysis due to collinearity issues with other variables. For the age group 80+, the following variables were found to have perfect prediction and were subsequently omitted from the analysis. The variable socialstigmascore_1 with a value of 1 predicts failure perfectly, and with a value of 0, it predicts success perfectly. These cases were omitted, and the outcome data for 1 observation (in each case) were not used. The variable w2c_rsex with a value of 1 predicts success perfectly. This variable was omitted, and the outcome data for 10 observations were not used. The variable w2c_bd_mar with a value of 2 predicts success perfectly. This variable was omitted, and the outcome data for 1 observation was not used. The variable w1c_bd_educ4 with a value of 1 predicts success perfectly. This variable was omitted, and the outcome data for 1 observation was not used. The variable w1c_ep_employed with a value of 0 predicts success perfectly. This variable was omitted, and the outcome data for 4 observations were not used. The variable w2c_hhsize with a value of 1 predicts success perfectly. This variable was omitted, and the outcome data for 1 observation was not used. Additionally, the outcome variable w2c_hhsize > 0 was found to perfectly predict the data. | 0.406 | 1.19 (0.36-3.99) | 0.777 | 0.02 (0.00-2.68) | 0.117 |  |  |

#### **Table A4 Association between anticipated stigma and ART uptake among PLWH by age group**

|  | **Age 40-49** | | **Age 50-59** | | **Age 60-69** | | **Age 70-79** | | **Age 80+** | |
| --- | --- | --- | --- | --- | --- | --- | --- | --- | --- | --- |
| **ART Uptake** | **Odds ratio (95% CI)** | **P-value** | **Odds ratio (95% CI)** | **P-value** | **Odds ratio (95% CI)** | **P-value** | **Odds ratio (95% CI)** | **P-value** | **Odds ratio (95% CI)** | **P-value** |
| 0 | REF | REF | REF | REF | REF | REF | REF | REF | REF | REF |
| 1.Anticipated stigma | 1.13 (0.33-3.85) | 0.841 | 0.94 (0.37-2.35) | 0.894 | 3.43 (1.29-9.11) | 0.013* | 6.71 (0.26-170.00) | 0.248 |  |  |
| **Sex** |  |  |  |  |  |  |  |  |  |  |
| Male | REF | REF | REF | REF | REF | REF | REF | REF | REF | REF |
| Female | 1.46 (0.57-3.72) | 0.434 | 1.18 (0.54-2.55) | 0.679 | 1.11 (0.42-2.95) | 0.831 | 3.00 (0.21-43.64) | 0.422 |  |  |
| **Marital status** |  |  |  |  |  |  |  |  |  |  |
| Never married | REF | REF | REF | REF | REF | REF | REF | REF | REF | REF |
| Separated or divorced | 2.19 (0.65-7.39) | 0.206 | 0.86 (0.24-3.04) | 0.817 | 0.76 (0.07-8.70) | 0.828 | 0.63 (0.01-70.17) | 0.846 |  |  |
| Widowed | 2.31 (0.60-8.98) | 0.225 | 4.14 (1.05-16.23) | 0.042* | 1.50 (0.13-17.51) | 0.744 | 6.11 (0.29-130.43) | 0.246 |  |  |
| Currently Married or Living w/ Partner | 1.70 (0.55-5.28) | 0.358 | 1.58 (0.41-6.08) | 0.503 | 4.13 (0.34-49.61) | 0.264 | 8.31 (0.35-195.22) | 0.189 |  |  |
| **Education** |  |  |  |  |  |  |  |  |  |  |
| No formal | REF | REF | REF | REF | REF | REF | REF | REF | REF | REF |
| Some primary (1-7 years) | 1.32 (0.39-4.49) | 0.658 | 0.52 (0.22-1.21) | 0.129 | 1.14 (0.47-2.77) | 0.776 | 0.89 (0.10-7.93) | 0.916 |  |  |
| Secondary or more (8+ years) | 1.27 (0.37-4.40) | 0.703 | 0.81 (0.31-2.12) | 0.671 | 1.73 (0.42-7.16) | 0.452 | 1 (empty) |  |  |  |
| **Employment** |  |  |  |  |  |  |  |  |  |  |
| Unemployed | REF | REF | REF | REF | REF | REF | REF | REF | REF | REF |
| Employed (part or full time) | 0.61 (0.25-1.47) | 0.268 | 0.51 (0.23-1.13) | 0.097 | 1.34 (0.33-5.46) | 0.687 | 0.06 (0.00-1.76) | 0.103 |  |  |
| Retired | 0.75 (0.11-4.93) | 0.761 | 0.52 (0.07-3.80) | 0.521 | 0.36 (0.14-0.93) | 0.035* | 2.04 (0.19-22.01) | 0.557 |  |  |
| **Household size** |  |  |  |  |  |  |  |  |  |  |
| Living alone | REF | REF | REF | REF | REF | REF | REF | REF | REF | REF |
| Living with one other person | 2.587 (0.44-15.10) | 0.291 | 1.95 (0.42-9.06) | 0.396 | 0.27 (0.05-1.56) | 0.142 | 1 (empty) |  |  |  |
| Living in 3-6 person household | 1.55 (0.43-5.61) | 0.503 | 1.21 (0.43-3.42) | 0.722 | 0.32 (0.07-1.55) | 0.157 | 1 (empty) |  |  |  |
| Living in 7+ person household | 0.85 (0.21-3.44) | 0.817 | 1.06 (0.34-3.36) | 0.918 | 0.11 (0.02-0.58) | 0.009** | 4.45 (0.48-41.60) | 0.190 |  |  |
| **Wave 2 wealth asset index** |  |  |  |  |  |  |  |  |  |  |
| Lower | REF | REF | REF | REF | REF | REF | REF | REF | REF | REF |
| Index 2 | 0.99 (0.34-2.91) | 0.990 | 1.84 (0.73-4.64) | 0.199 | 1.90 (0.60-6.06) | 0.279 | 0.08 (0.00-3.71) | 0.199 |  |  |
| Index 3 | 2.24 (0.22-22.63) | 0.495 | 2.99 (0.55-16.13) | 0.204 | 7.34 (0.73-74.11) | 0.091 | 1 (empty) |  |  |  |
| Index 4 | 0.80 (0.19-3.40) | 0.767 | 1.41 (0.45-4.36) | 0.553 | 1.60 (0.42-6.00) | 0.490 | 1 (empty) |  |  |  |
| Higher | 4.22 (0.87-20.51) | 0.074 | 1.40 (0.54-3.65) | 0.493 | 0.95 (0.30-3.03) | 0.935 | 0.10 (0.00-3.53) | 0.208 |  |  |

*p<0.05, **p<0.01. For age group 70-79, several variables were found to have perfect prediction, leading to their omission from the analysis. The variable w2c_hhsize with a value of 0 predicts success perfectly. This case was omitted, and the outcome data for 6 observations were not used. Additionally, when w2c_hhsize had a value of 1, it also predicts success perfectly, leading to the omission of another 6 observations. The variable w2c_wealthindex with a value of 3 predicts success perfectly. This case was omitted, and the outcome data for 5 observations were not used. Similarly, when w2c_wealthindex had a value of 4, it also predicts success perfectly, leading to the omission of another 6 observations. The variable w1c_bd_educ4 with a value of 3 predicts failure perfectly. This case was omitted, and the outcome data for 1 observation was not used. w2c_hhsize, this variable was omitted due to collinearity, indicating that it may be highly correlated with other variables in the model, making its inclusion redundant or problematic for estimation. For age group 80+, several variables were found to have perfect prediction, resulting in their omission from the analysis. The variable secrete with a value of 0 predicts success perfectly. This case was omitted, and the outcome data for 3 observations were not used. The variable w2c_rsex with a value of 1 predicts success perfectly. This case was omitted, and the outcome data for 9 observations were not used. The variable w2c_bd_mar with a value of 2 predicts success perfectly. This case was omitted, and the outcome data for 1 observation was not used. The variable w1c_bd_educ4 with a value of 1 predicts success perfectly. This case was omitted, and the outcome data for 1 observation was not used. The variable w1c_ep_employed with a value of 0 predicts success perfectly. This case was omitted, and the outcome data for 4 observations were not used. The variable w2c_hhsize with a value of 1 predicts success perfectly, and with a value of 2, it predicts failure perfectly. These cases were omitted, and the outcome data for 1 observation was not used for each value. Additionally, the outcome variable w2c_wealthindex with a condition <= 0 predicts data perfectly, but no specific observations were omitted in this case.

#### **Table A5 Association between social stigma and self-report HIV testing among older adults by sex**

|  | **Male** | | **Female** | |
| --- | --- | --- | --- | --- |
| **Ever tested** | **Odds ratio (95% CI)** | **P-value** | **Odds ratio (95% CI)** | **P-value** |
| **Social Stigma Score** |  |  |  |  |
| 0 | REF | REF | REF | REF |
| 1 | 0.58 (0.42-0.79) | 0.001** | 0.71 (0.53-0.96) | 0.026* |
| 2 | 0.64 (0.43-0.96) | 0.029* | 0.58 (0.41-0.83) | 0.003** |
| 3 | 0.44 (0.26-0.76) | 0.003** | 0.71 (0.41-1.26) | 0.247 |
| **Age group** |  |  |  |  |
| 40-49 | REF | REF | REF | REF |
| 50-59 | 1.09 (0.68-1.73) | 0.726 | 0.55 (0.35-0.87) | 0.011* |
| 60-69 | 0.83 (0.52-1.31) | 0.421 | 0.59 (0.36-0.95) | 0.032* |
| 70-79 | 0.62 (0.38-1.02) | 0.062 | 0.30 (0.18-0.51) | 0.000*** |
| 80+ | 0.45 (0.26-0.76) | 0.003** | 0.21 (0.12-0.37) | 0.000*** |
| **Marital Status** |  |  |  |  |
| Never Married | REF | REF | REF | REF |
| Separated or divorced | 1.74 (1.07-2.82) | 0.026* | 2.18 (1.28-3.69) | 0.004** |
| Widowed | 2.09 (1.31-3.33) | 0.002** | 1.87 (1.18-2.95) | 0.008** |
| Currently Married or Living w/ Partner | 2.99 (2.02-4.44) | 0.000*** | 1.99 (1.25-3.17) | 0.004** |
| **Education** |  |  |  |  |
| No formal | REF | REF | REF | REF |
| Some primary (1-7 years) | 1.18 (0.90-1.55) | 0.240 | 1.27 (0.99-1.63) | 0.065 |
| Secondary or more (8+ years) | 1.36 (0.94-1.96) | 0.103 | 1.25 (0.86-1.83) | 0.248 |
| **Employment** |  |  |  |  |
| Unemployed | REF | REF | REF | REF |
| Employed (part or full time) | 1.24 (0.87-1.77) | 0.228 | 0.90 (0.63-1.29) | 0.578 |
| Retired | 0.93 (0.70-1.23) | 0.598 | 0.88 (0.68-1.15) | 0.359 |
| **Household Size** |  |  |  |  |
| Living alone | REF | REF | REF | REF |
| Living with one other person | 0.51 (0.32-0.81) | 0.004** | 1.60 (0.97-2.63) | 0.065 |
| Living in 3-6 person household | 0.65 (0.43-0.98) | 0.039* | 1.42 (0.96-2.11) | 0.081 |
| Living in 7+ person household | 0.53 (0.34-0.82) | 0.004** | 0.87 (0.58-1.30) | 0.497 |
| **Wave 2 Wealth Index Asset** |  |  |  |  |
| Lower | REF | REF | REF | REF |
| Index=2 | 1.11 (0.80-1.53) | 0.537 | 1.38 (1.03-1.85) | 0.033* |
| Index=3 | 1.08 (0.64-1.83) | 0.767 | 0.75 (0.48-1.17) | 0.206 |
| Index=4 | 1.05 (0.71-1.56) | 0.816 | 1.07 (0.75-1.53) | 0.707 |
| Higher | 1.65 (1.14-2.38) | 0.007** | 1.21 (0.88-1.66) | 0.239 |

*p<0.05, **p<0.01, ***p<0.001

#### **Table A6 Association between anticipated stigma and self-report HIV testing among older adults by sex**

|  | **Male** | | **Female** | |
| --- | --- | --- | --- | --- |
| **Ever tested** | **Odds ratio (95% CI)** | **P-value** | **Odds ratio (95% CI)** | **P-value** |
| 0 | REF | REF | REF | REF |
| **1. Anticipated stigma** | 1.30 (0.95-1.79) | 0.100 | 1.10 (0.82-1.48) | 0.508 |
| **Age group** |  |  |  |  |
| 40-49 | REF | REF | REF | REF |
| 50-59 | 1.05 (0.66-1.68) | 0.822 | 0.54 (0.34-0.86) | 0.009** |
| 60-69 | 0.80 (0.50-1.27) | 0.346 | 0.56 (0.35-0.92) | 0.021* |
| 70-79 | 0.59 (0.36-0.97) | 0.038* | 0.29 (0.17-0.48) | 0.000*** |
| 80+ | 0.41 (0.24-0.70) | 0.001** | 0.19 (0.11-0.32) | 0.000*** |
| **Marital status** |  |  |  |  |
| Never married | REF | REF | REF | REF |
| Separated or divorced | 1.76 (1.09-2.85) | 0.021* | 2.19 (1.29-3.71) | 0.004** |
| Widowed | 2.11 (1.33-3.35) | 0.001** | 1.92 (1.21-3.02) | 0.005** |
| Currently Married or Living w/ Partner | 3.06 (2.07-4.53) | 0.000*** | 2.07 (1.30-3.29) | 0.002** |
| **Education** |  |  |  |  |
| No formal | REF | REF | REF | REF |
| Some primary (1-7 years) | 1.17 (0.89-1.53) | 0.253 | 1.26 (0.98-1.62) | 0.068 |
| Secondary or more (8+ years) | 1.39 (0.97-2.01) | 0.076 | 1.23 (0.85-1.80) | 0.275 |
| **Employment** |  |  |  |  |
| Unemployed | REF | REF | REF | REF |
| Employed (part or full time) | 1.27 (0.89-1.80) | 0.191 | 0.91 (0.64-1.3) | 0.623 |
| Retired | 0.91 (0.69-1.22) | 0.539 | 0.89 (0.69-1.15) | 0.379 |
| **Household Size** |  |  |  |  |
| Living alone | REF | REF | REF | REF |
| Living with one other person | 0.53 (0.34-0.85) | 0.007** | 1.55 (0.95-2.55) | 0.082 |
| Living in 3-6 person household | 0.67 (0.45-1.01) | 0.053* | 1.36 (0.92-2.01) | 0.127 |
| Living in 7+ person household | 0.54 (0.35-0.84) | 0.006** | 0.83 (0.55-1.24) | 0.365 |
| **Wave 2 wealth asset index** |  |  |  |  |
| Lower | REF | REF | REF | REF |
| Index 2 | 1.13 (0.82-1.56) | 0.462 | 1.41 (1.05-1.89) | 0.022* |
| Index 3 | 1.09 (0.65-1.83) | 0.755 | 0.77 (0.49-1.20) | 0.243 |
| Index 4 | 1.08 (0.73-1.60) | 0.708 | 1.09 (0.76-1.55) | 0.647 |
| Higher | 1.71 (1.19-2.46) | 0.004** | 1.24 (0.91-1.71) | 0.177 |

*p<0.05, **p<0.01, ***p<0.001

#### **Table A7 Association between social stigma and ART uptake among PLWH by sex**

|  | **Male** | |  | **Female** | |
| --- | --- | --- | --- | --- | --- |
| **ART uptake** | **Odds ratio (95% CI)** | **P-value** |  | **Odds ratio (95% CI)** | **P-value** |
| **socialstigmascore_1** |  |  |  |  |  |
| 0 | REF | REF |  | REF | REF |
| 1 | 0.57 (0.22-1.45) | 0.239 |  | 0.95 (0.40-2.27) | 0.903 |
| 2 | 0.70 (0.22-2.19) | 0.537 |  | 0.28 (0.10-0.83) | 0.022* |
| 3 | 0.28 (0.08-0.97) | 0.046* |  | 0.87 (0.08-9.18) | 0.905 |
| **Age group** |  |  |  |  |  |
| 40-49 | REF | REF |  | REF | REF |
| 50-59 | 0.83 (0.36-1.92) | 0.658 |  | 0.88 (0.42-1.84) | 0.728 |
| 60-69 | 2.61 (1.01-6.78) | 0.048* |  | 0.67 (0.28-1.62) | 0.374 |
| 70-79 | 1.88 (0.52-6.79) | 0.335 |  | 0.93 (0.25-3.48) | 0.915 |
| 80+ | 1 (empty) |  |  | 0.58 (0.09-3.78) | 0.569 |
| **Marital Status** |  |  |  |  |  |
| Never Married | REF | REF |  | REF | REF |
| Separated or divorced | 1.37 (0.46-4.10) | 0.570 |  | 0.82 (0.29-2.30) | 0.709 |
| Widowed | 5.90 (1.45-24.00) | 0.013* |  | 2.02 (0.74-5.56) | 0.171 |
| Currently Married or Living w/ Partner | 2.67 (0.94-7.55) | 0.064 |  | 1.82 (0.61-5.44) | 0.287 |
| **Education** |  |  |  |  |  |
| No formal | REF | REF |  | REF | REF |
| Some primary (1-7 years) | 1.31 (0.60-2.88) | 0.502 |  | 0.55 (0.28-1.06) | 0.072 |
| Secondary or more (8+ years) | 1.28 (0.53-3.07) | 0.581 |  | 0.81 (0.35-1.85) | 0.609 |
| **Employment** |  |  |  |  |  |
| Unemployed | REF | REF |  | REF | REF |
| Employed (part or full time) | 1.22 (0.55-2.74) | 0.627 |  | 0.34 (0.18-0.68) | 0.002** |
| Retired | 0.45 (0.17-1.18) | 0.106 |  | 0.62 (0.26-1.52) | 0.297 |
| **Household Size** |  |  |  |  |  |
| Living alone | REF | REF |  | REF | REF |
| Living with one other person | 1.05 (0.28-3.96) | 0.943 |  | 1.71 (0.46-6.29) | 0.421 |
| Living in 3-6 person household | 0.70 (0.26-1.87) | 0.481 |  | 1.39 (0.51-3.81) | 0.518 |
| Living in 7+ person household | 0.30 (0.10-0.90) | 0.032* |  | 0.93 (0.33-2.59) | 0.886 |
| **Wave 2 wealth Asset index** |  |  |  |  |  |
| Lower | REF | REF |  | REF | REF |
| Index 2 | 1.13 (0.48-2.64) | 0.774 |  | 1.14 (0.55-2.37) | 0.725 |
| Index 3 | 7.09 (0.80-62.59) | 0.078 |  | 2.02 (0.53-7.70) | 0.304 |
| Index 4 | 1.90 (0.56-6.41) | 0.302 |  | 0.76 (0.31-1.87) | 0.550 |
| Higher | 1.34 (0.51-3.45) | 0.546 |  | 1.23 (0.56-2.74) | 0.606 |

*p<0.05, **p<0.01

The variable age group 80+ for male predicts success perfectly. As a result, this case was omitted from the analysis, and the outcome data for 11 observations were not used.

####

#### **Table A8 Association between anticipated stigma and ART uptake among PLWH by sex**

|  | **Male** | | **Female** | |
| --- | --- | --- | --- | --- |
| **ART uptake** | **Odds ratio (95% CI)** | **P-value** | **Odds ratio (95% CI)** | **P-value** |
| 0 | REF | REF | REF | REF |
| 1. Anticipated stigma | 2.35 (1.07-5.14) | 0.033* | 1.05 (0.48-2.28) | 0.912 |
| **Age group** |  |  |  |  |
| 40-49 | REF | REF | REF | REF |
| 50-59 | 0.91 (0.39-2.09) | 0.818 | 0.83 (0.40-1.74) | 0.620 |
| 60-69 | 2.61 (1.00-6.79) | 0.049* | 0.61 (0.25-1.46) | 0.263 |
| 70-79 | 1.81 (0.49-6.60) | 0.371 | 0.78 (0.21-2.82) | 0.701 |
| 80+ | 1 (empty) |  | 0.47 (0.07-3.00) | 0.423 |
| **Marital status** |  |  |  |  |
| Never married | REF | REF | REF | REF |
| Separated or divorced | 1.36 (0.47-3.98) | 0.573 | 0.93 (0.33-2.56) | 0.882 |
| Widowed | 6.28 (1.55-25.41) | 0.010* | 2.25 (0.83-6.13) | 0.112 |
| Currently Married or Living w/ Partner | 2.78 (0.99-7.81) | 0.053* | 2.07 (0.70-6.13) | 0.190 |
| **Education** |  |  |  |  |
| No formal | REF | REF | REF | REF |
| Some primary (1-7 years) | 1.21 (0.55-2.64) | 0.634 | 0.52 (0.27-0.99) | 0.048* |
| Secondary or more (8+ years) | 1.35 (0.56-3.27) | 0.499 | 0.72 (0.32-1.64) | 0.441 |
| **Employment** |  |  |  |  |
| Unemployed | REF | REF | REF | REF |
| Employed (part or full time) | 1.45 (0.64-3.30) | 0.371 | 0.36 (0.19-0.70) | 0.003** |
| Retired | 0.48 (0.18-1.25) | 0.131 | 0.71 (0.29-1.70) | 0.437 |
| **Household size** |  |  |  |  |
| Living alone | REF | REF | REF | REF |
| Living with one other person | 1.09 (0.29-4.13) | 0.899 | 1.75 (0.48-6.40) | 0.396 |
| Living in 3-6 person household | 0.70 (0.26-1.87) | 0.477 | 1.33 (0.49-3.58) | 0.575 |
| Living in 7+ person household | 0.30 (0.10-0.92) | 0.035* | 0.89 (0.32-2.45) | 0.820 |
| **Wave 2 wealth asset index** |  |  |  |  |
| Lower | REF | REF | REF | REF |
| Index 2 | 1.12 (0.48-2.61) | 0.793 | 1.17 (0.57-2.43) | 0.665 |
| Index 3 | 5.63 (0.65-48.57) | 0.116 | 2.07 (0.55-7.86) | 0.284 |
| Index 4 | 2.03 (0.61-6.84) | 0.251 | 0.80 (0.33-1.95) | 0.628 |
| Higher | 1.27 (0.49-3.26) | 0.625 | 1.20 (0.55-2.63) | 0.644 |

*p<0.05, **p<0.01

The variable age group 80+ for male predicts success perfectly. As a result, this case was omitted from the analysis, and the outcome data for 11 observations were not used.
